# Do interventions to promote walking in groups increase physical activity? A meta-analysis

**DOI:** 10.1186/1479-5868-10-18

**Published:** 2013-02-06

**Authors:** Aikaterini Kassavou, Andrew Turner, David P French

**Affiliations:** 1 Centre for Primary Care and Public Health, Queen Mary, University of London, London, UK; 2 Applied Research Centre in Health & Lifestyle Interventions, Coventry University, Coventry, UK; 3 School of Psychological Sciences, University of Manchester, Manchester, UK

**Keywords:** Walking in groups, Interventions, Physical activity, Systematic review, Meta-analysis

## Abstract

**Objective:**

Walking groups are increasingly being set up but little is known about their efficacy in promoting physical activity. The present study aims to assess the efficacy of interventions to promote walking in groups to promoting physical activity within adults, and to explore potential moderators of this efficacy.

**Method:**

Systematic literature review searches were conducted using multiple databases. A random effect model was used for the meta-analysis, with sensitivity analysis.

**Results:**

The effect of the interventions (19 studies, 4 572 participants) on physical activity was of medium size (d = 0.52), statistically significant (95%CI 0.32 to 0.71, p < 0.0001), and with large fail-safe of N = 753. Moderator analyses showed that lower quality studies had larger effect sizes than higher quality studies, studies reporting outcomes over six months had larger effect sizes than studies reporting outcomes up to six months, studies that targeted both genders had higher effect sizes than studies that targeted only women, studies that targeted older adults had larger effect sizes than studies that targeted younger adults. No significant differences were found between studies delivered by professionals and those delivered by lay people.

**Conclusion:**

Interventions to promote walking in groups are efficacious at increasing physical activity. Despite low homogeneity of results, and limitations (e.g. small number of studies using objective measures of physical activity, publication bias), which might have influence the findings, the large fail-safe N suggests these findings are robust. Possible explanations for heterogeneity between studies are discussed, and the need for more investigation of this is highlighted.

## Background

The health benefits of physical activity are well documented [1,2]. Governmental recommendations suggest that adults and older adults should participate in at least 150 minutes of moderate or vigorous activity per week, in order to promote and maintain health [3,4]. However, most adults do not currently achieve this [5,6]. Thus, promoting physical activity as an integral part of lifestyle has become a central aim of public health policy [3,4].

Moderate intensity physical activity includes walking, which is a popular, accessible and acceptable form of activity particularly among populations who are the most physically inactive [7]. Walking also does not require special equipment and has low risk of injuries. Further, a meta-analysis of relevant research found that increased walking led to increased fitness, decreased body weight, Body Mass Index, percentage body fat and resting diastolic blood pressure in previously sedentary adults [8].

Group walking has become an increasingly popular form of promoting physical activity in many countries, especially among sedentary people and people with chronic diseases. For example, Walking for Health (WfH) is an initiative, which has established group walks across England with the aim to encourage more people to be physically active. During 2011 they ran 522 walk schemes across England with more than 57 585 walkers [9]. Likewise, the American Volkssport Association reports more than 300 walking clubs in the United States [10].

Several systematic literature reviews have described the efficacy of interventions to promote physical activity in different populations [11]. Despite this, no review exists on the efficacy of interventions to promote walking in groups in promoting physical activity. Currently, the best source of evidence on the efficacy of walking interventions is the 2007 systematic review by Ogilvie and colleagues [12]. This review included interventions to promote walking in groups under the umbrella of either group based walking interventions (as led walks) or community interventions (as formation of walking groups). However, in this review there is no distinct category of interventions to promote walking in groups. For example under group based interventions there are not only interventions where the behaviour is implemented in groups [13] but also interventions delivered to groups, as education sessions, but where the walking is not enacted in groups [14]. In the present review we focus solely on interventions to promote walking in groups, which are interventions where participants walk collectively in organised walking groups and thereby provide support relationships for behaviour change.

Given that previous research does not provide clear evidence of the efficacy of walking in groups interventions, the aim of the present study was to systematically appraise the efficacy of these interventions to promote physical activity for adults. Sensitivity analysis explored whether the quality of studies affected estimates of effect size. Secondary aims were to assess whether the efficacy of these interventions varied according to duration of follow-up measurements, participants’ gender, participants’ age and the person delivering the intervention.

## Methods

### Eligibility criteria

Randomized Control Trials (RCTs), non Randomized Control Trials (non-RCTs) and pre-post studies were included. Only studies with objective or self-reported measures of physical activity were eligible for inclusion. The outcome was assessed in terms of the frequency, duration or distance of physical activity performed. Studies with adults over 18 years old were included in the review. Only studies reported in English were eligible, due to lack of resources available for translation.

The following types of studies were included: (a) interventions in which people walk collectively in groups. In these studies walking in group might be the only component of the behavior change interventions or might be one component of wider behavior change intervention, (b) interventions where participants walk with or without leaders’ physical presence, and (c) interventions where people are allowed to choose a partner to walk with (i.e. group is defined as two people or more).

Studies were excluded for the following reasons: (a) interventions to promote walking in groups where the dependent variable measured was not physical activity behaviour, but the outcomes of the behaviour, such as heart rate, energy loss, exercise intensity, endurance and functional capacity, tolerance, energy loss, balance [15-17], (b) interventions which included walking groups as one component of a wider physical activity intervention but measured the total level of physical activity, not only effects of walking in groups [18-20], (c) interventions when group walking is taking place in a laboratory using physical activity equipment such as corridors, treadmill or mat walking [21], (d) interventions when people have a collective goal but walk individually/independently from each other [22], (e) interventions to promote walking in groups when compared the outcomes of one walking group with another walking group and not with a control group or did not have baseline measures for each of the intervention groups [23,24], (f) studies when participants walk accompanied by the researcher but not with other participants, (g) group based walking interventions when participants did not *enact* walking behavior collectively [25,26], (h) studies where participants exercise in groups or complete specified levels of other type of physical activities in groups in order to provide social support to each other but they do not walk in groups [27].

### Information sources

Studies were identified through searches in the following electronic databases: Academic search complete, PsycINFO, Medline, CINAHL with full text, AMED, SportDiscus and Scopus (from January 1980 to March 2012).

### Search

Search terms varied depending upon the database being searched, but in all cases the following terms were used: walking in groups, group walking, led walk, walking club, club walking, group physical activity, group exercise, interventions, pedometer interventions, and program (see Additional file 1).

All potentially relevant articles were screened by abstract and where appropriate articles were retrieved in full text for detailed examination. References from review articles [11,12,28-30] on walking and physical activity interventions were screened for relevant studies. Forward and backward citation searches of included papers were made. Authors of studies that satisfied the inclusion criteria were contacted by email asking for unpublished study data in order to identify grey literature. The first author performed the search and the selection of the studies.

### Data extraction process

The first author and a second researcher extracted^a^ the data independently, in a standardized manner using a coding frame (coding frame is provided in Additional file 2). Any discrepancies were resolved by discussion. Good agreement between coders was achieved (between κ = 0.84 and κ = 0.98).

### Synthesis of results

The effect size used was the Standardized Mean Difference also known as Cohen’s d [31]. When physical activity was measured on more than two occasions, baseline and the last follow up measurement were extracted. Means and pooled standard deviation were used to calculate effect sizes. Data were analyzed using the meta-analysis program of Schwarzer [32]. A random effect model was used. Heterogeneity was assessed using Q coefficients, which assess between-studies variabilities. The *Q* test is computed by summing the squared deviations of each study’s effect estimate from the overall effect estimate, weighting the contribution of each study by its inverse variance [33]. Fail-safe N was calculated to explore the extent to which the “file drawer problem” may have affected study results [34]. The Fail-safe N specifies the number of hypothetical studies showing a zero effect that required to be included in the meta-analysis for the relationship between independent and dependent variables to become statistically non significant.

An assessment of the quality of the included studies was made using the guide by the Cochrane Consumers and Communication Review Group [35], to explore whether poor quality studies biased the results. Studies, which satisfied a quality criterion were awarded a point “*yes*”. If the criterion was not satisfied or details on the process followed to satisfy the criterion were not provided, then no points were given (more details is provided in Additional file 3). Studies scoring at least 4 points (i.e. “yes” on at least half the criteria) were classified as higher quality and studies scoring 0–3 points as lower quality. Quality criteria and assessment can be found in Table 1.

**Table 1:** Sensitivity analysis: criteria and assessment of the quality of included studies based on Study Quality Guide 2011 by Cochrane Consumers and Communication Review Group

**Criteria**	**Randomization**	**Allocation**	**Blinding**	**Baseline comparability**	**Follow up**	**Valid measures**	**Ethical approval**	**Informed consent**	**Score**
**Studies**									
[36] Kriska 1986	Yes	Yes	Ncl	Yes	Yes	Yes	Yes	Yes	7
[37] McAuley 1994	Yes	Ncl	Ncl	Ncl	Yes	No	Yes	Yes	4
[38] Rogers 1997	No	No	No	No	No	Yes	Yes	Yes	3
[39] Sullivan 1998	Yes	Ncl	No	No	No	Yes	Yes	Yes	4
[40] Resnick 2002	Yes	Ncl	Ncl	Yes	Yes	Yes	Yes	Yes	6
[41] Coull 2004	Yes	Yes	Yes	Yes	Yes	No	Yes	Yes	7
[13] Fisher 2004	Yes	Yes	No	Yes	Yes	No	Yes	Yes	6
[42] Nguyen 2002	No	No	No	No	Yes	No	Ncl	Ncl	1
[43] Staten 2005	No	No	No	No	Yes	No	Ncl	Yes	2
[44] Banks-Wallace 2007	No	No	No	No	No	Yes	Yes	Yes	3
[45] Hogue 2007	No	No	No	No	Yes	No	Yes	Yes	3
[46] Estabrooks 2008	Ncl	No	No	No	No	No	Ncl	Ncl	0
[47] Jancey 2008	Ncl	No	No	Yes	No	Yes	Yes	Yes	4
[48] Krieger 2009	No	No	No	No	Yes	Ncl	Ncl	Ncl	1
[49] Zoellner 2010	No	No	No	No	Yes	Yes	Yes	Yes	4
[50] Takeda 2011	No	No	No	Yes	Yes	Yes	Yes	Yes	5
[51] Lee 2012	Yes	Yes	Yes	Yes	No	Yes	Yes	Yes	7
[52] Maki 2012	Ncl	Yes	Yes	No	Yes	Yes	Yes	Yes	6
[53] Thomas 2012	Yes	Yes	Yes	Yes	Yes	Yes	Yes	Yes	8

Yes = satisfied quality criterion, No = do not satisfy quality criteria, Ncl = information provided not clear.

Sensitivity analysis and moderator analyses were performed by breaking down the data into two subsets with respect to a theoretically relevant variable. In order to classify as a moderator, the following requirements had to be met: (a) the effect size varies from subset to subset, and (b) the residual variance averages lower in the subsets than for the data as a whole [54]. Pair wise z tests were used to assess whether there was any statistically significant differences in effect size estimates for interventions that:

measure outcomes up to six months post baseline (0–6 months) vs measured outcome over six months after baseline (>6 months). When interventions had different measurements at baseline and follow up, only the follow up measurements were used for this analysis.

differ on participants’ gender. Originally we intended to compare whether there is any difference between women’s groups and men’s groups but the lack of interventions that targeted only men led us to change the moderator analysis. Instead, all studies were categorised into two groups, those that targeted only women and those that targeted both genders.

differ on participants’ age. Studies were categorised into two groups, younger adults (≤60 years old) and older adults (>61 years old).

were delivered by a lay person or by the research or other professionals.

## Results

**Figure 1:**
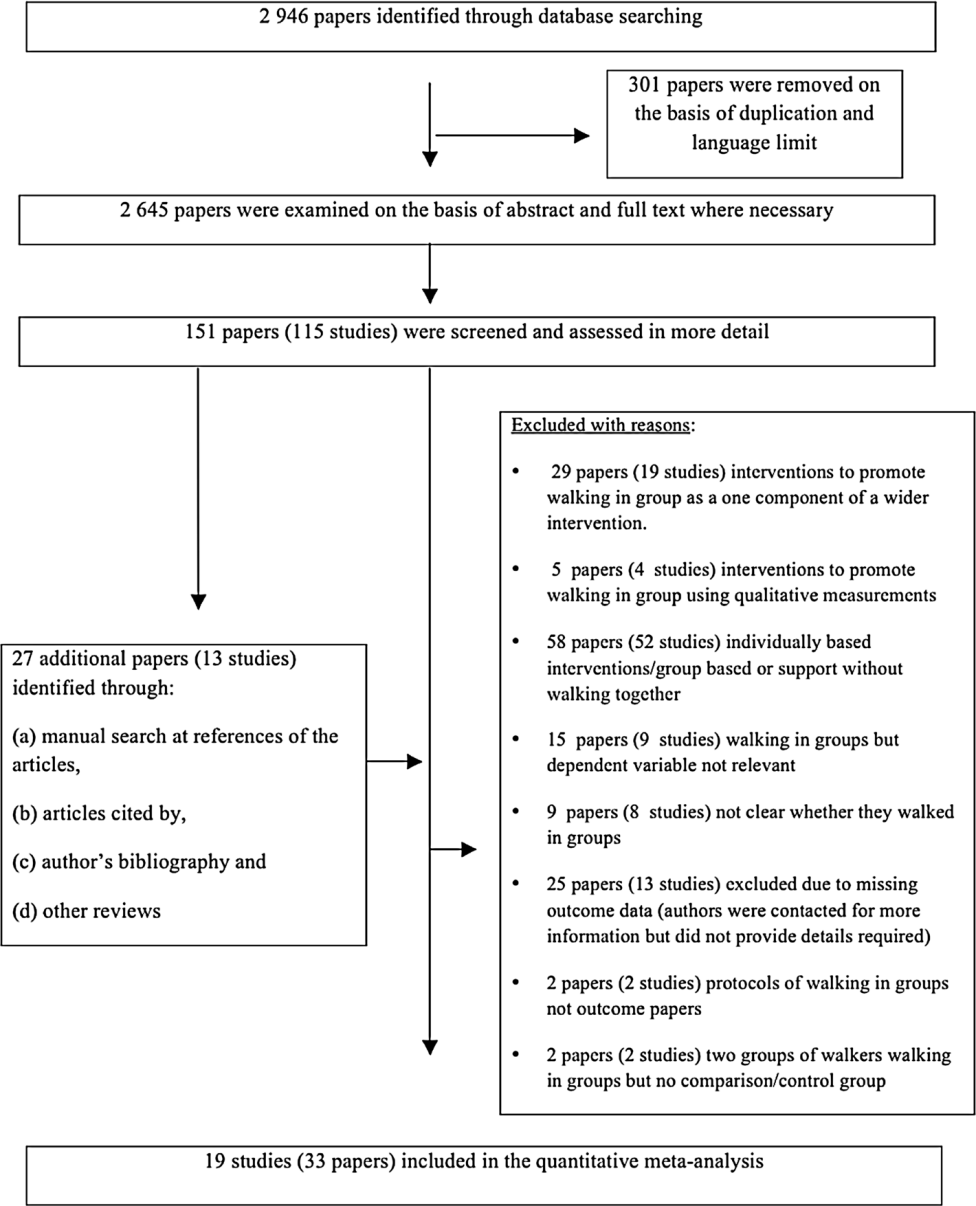
Flow chart of searches conducted and study selection.

The systematic electronic search retrieved 2 946 unique papers. Based on examination of these papers, 151 papers describing 115 unique studies were retrieved in full text for detailed examination. Further searches and screening against inclusion criteria concluded in 19 studies (presented in 33 papers), which were included for quality appraisals and analysis in this review (see flow diagram in Figure 1).

Nine of these 19 studies included were RCTs [13,36,37,39-41,51-53], six studies were non-RCTs [38,45-47,49,50] and four had pre-post designs [42-44,48]. Seventeen studies were community based [13,36-38,40,42-53] and two were hospital-based [39,41]. From the seventeen community-based studies, one study was in care retirementcommunity [40], one was in a centre for older people [53], one was based in a church [45], three were neighborhood based [13,44,47] and the rest eleven [36-38,42,43,46,48-52] were based in the general community. Based on available data, the overall mean age of participants was 59.8 years (ranged from 44 to 88).

Eleven studies were walking in groups only interventions [13,36,37,42,44,47,49-53], four studies involved walking in groups and other types of PA [38,45,46,48], and four studies contained walking in groups and supportive patient education program on chronic diseases (e.g. self-management) or other lifestyle behaviors [39-41,43]. Thirteen studies were conducted in US [13,36-40,43-46,48,49,51], one in UK [41], one in Canada [42], one in Australia [47], two in Japan [50,52] and one in China [53] (more details are given in Table 2).

**Table 2:** Summary of characteristics of included studies with theoretically relevant variables for conducting moderator analyses

**Studies by author and date order**	**Group characteristics**	**Setting**	**Design**	**Delivered mode**	**Duration and timing of walking in groups intervention**	**Assessment of physical activity**	**Measurements**	**Gender**	**Targeted age**	**Delivered by**
[36] Kriska et al. 1986, [56] Pereira et al. 1998	Postmenopausal women (n = 229) Mean age: 57.5	Community	RCT	Training sessions Newsletter	Twice per week with goal to reach 60 minutes per session, for 8 weeks	Pedometers Paffenbarger questionnaire (blocks walked)* Monthly log sheets Multiple item scale	Baseline 1 year 2 years	Only women	Middle age adults	Lay people
[37] McAuley et al. 1994	Adults (n = 228) Mean age: 54.52	Community	RCT	Training sessions	Three times per week starting with 10-15 min and progressing to 40 min, for 5 months.	Diaries	Baseline 5 months	Both genders	Middle age adults	Experts with the research area and the population
[57] McAuley et al. 2003										
[38] Rogers 1997	African American women (n = 35) Mean age: 51.6	Community	Non-RCTs	Training session Discussion groups	Once per week for 60 to 90 minutes, for 5 weeks	7-days recall physical activity questionnaire developed by Steinhardt and Dishman (1989 modified by Blair 1984) * Walking calendars	Baseline 5 weeks 7 weeks	Only women	Middle age adults	Researcher
[39] Sullivan et al. 1998	Older adults with chronic diseases (n = 52) Mean age: 71.2	Hospital	RCT	Discussion group Self help manual for participants	Three times per week for approximately 90 minutes, for 8 weeks	AIMS physical activity subscale* Diaries Self report questions	Baseline 8 weeks 1 year	Both genders	Older adults	Researcher
[40] Resnick 2002	Older adults (n = 34) Mean age:88	Retirement community	RCT	One-to-one visit	Three times per week for 20 minutes, for 6 months.	YPAS (interviewed administered questionnaire) Diaries *	Baseline, 2 months 6 months	Only women	Older adults	Experts with the research area and the population
[41] Coull et al. 2004	Older adults with ischemic heart disease (n = 289) Mean age: 67.5	Hospital	RCT	Training sessions	Once per month for approx 2 hour, for 1 year	Questions about previous week’s physical activity	Baseline 1 year	Both genders	Older adults	Lay volunteers
[13] Fisher et al. 2004, [58] Fisher et al. 2004a, [59] Fisher et al. 2002, [60] Michael et al. 2009, [61] Rowland et al. 2004	Older adults (n = 527) Mean age: 74.5	Community (Neighbourhood)	RCT	Training sessions (for leaders) Discussion group Self help manual Monthly newsletter	Three times per week for approximately 1 hour, for 6 months	Multiple item scale (3 items measuring the frequency of neighbourhood walking activity)	Baseline 3 months 6 months	Both genders	Older adults	Lay people
[42] Nguyen et al. 2002, [55] Nguyen et al. 2005	Adults (n = 575) Mean age: 54.6	Community	Pre post study	Newsletter for walkers Training session for walk leaders Guide for walk leaders Walking kit	Two to three times per week for 12 months (duration of sessions not given)	Multiple item scale (duration and frequency of physical activity)	Baseline 12 months	Both genders	Middle age adults	Lay volunteers
[43] Staten et al. 2005	Adults at risk for chronic diseases (n = 432) Mean age: 52.7	Community	Pre post study	Self help manuals for walk leaders Training session on preventing chronic diseases	Three times per week for 1 year (duration of sessions not given)	MLPAQ	Baseline 12 weeks	Both genders	Middle age adults	Lay people
[44] Banks-Wallace et al. 2007	African American women at risk for CVD (n = 36) Mean age: 50.3	Community (Neighbourhood)	Pre post study	Discussion groups Self help manuals (Stanford Walking Kit)	Twice per week for 1 year (duration of sessions not given)	Pedometers * CAPSPAQ Diaries/ walking calendars	Baseline 12 months 18 months	Only women	Middle age adults	Researcher/ professional story teller
[62] Banks-Wallace et al. 2005										
[45] Hogue 2007	African American women (n = 46) Mean age: 46	Community (Church)	Non-RCTs	education meetings- sessions educational material remind calls	Daily for 10 weeks	Physical Activity Readiness Questionnaire (PAR-Q) Rapid Assessment of Physical Activity (RAPA I & RAPA II) Pedometer Six minute walk test Physical Activity Minutes Log Sheet * Buddy Support Log: minutes spent exercising with buddy	Baseline 10 weeks	Only women	Middle age adults	Research assistant (faculty members and graduate students) – did not participated as subjects
[46] Estabrooks et al. 2008	Adults (n = 380) Mean age: 46	Community	Non-RCTs	Not stated	8 weeks intervention	Two questions, which assesses moderate physical activity* Survey (centre for disease and prevention behavioral risk factor surveillance survey)	Baseline to 8 weeks, Baseline to 6 months 8 weeks to 6 months	Both genders	Middle age adults	Ncl
[63] Burke et al. 2010,										
[47] Jancey et al. 2008; [64] Jancey et al. 2008a, [65] Jancey et al. 2006, [66] Jancey et al. 2007, [67] Jancey et al. 2011	Older adults (n = 573) Mean age: 69.2	Community (Neighbourhood)	Non-RCTs	Training sessions Self help manuals	Twice per week started with 10 minutes and built up to 45 min and gradually increase intensity, for 6 months	IPAQ^1^	Baseline 3 months 6 months	Both genders	Older adults	Lay volunteers
[48] Krieger et al. 2009	Adults (n = 106) Mean age: ncl	Community	Pre – post study	Walk kit from California centre of physical activity	Five times per week for 1 hour (which varied depending on walker’s capacity)	Behavioural risk factor surveillance system questionnaire		Both genders	Middle age adults	Lay people^2^
[49] Zoellner et al. 2010	Adults (n = 112) Mean age: 44	Community	Non-RCTs	Training sessions Manual	Once per month for approx 90 min education session and walk throughout the week	Face-to-face administered questionnaire including past week physical activity recall Pedometer *	Baseline 3 months 6 months	Both genders	Middle age adults	Lay volunteers
[68] Zoellner et al. 2007										
[69] Zollenner et al. 2009										
[70] Zoellner et al. 2011										
[71] Powers 2007										
[50] Takeda 2011	Adults (n = 47) Mean age: 59.7	Community	Non-RCTs	Group instruction classes- lectures Newsletters	140 minutes sessions for 8 weeks (number of sessions not specified)	Pedometer with an accelerometer	Baseline 2 months 6 months	Both genders	Middle age adults	Staff
[51] Lee 2012	African American and Hispanic or Latina women (n = 322) Mean age: 45.7	Community	RCT	Sessions	6 sessions for a period of 6 months with the goal to gradually increase walking to recommended guidelines	IPAQ Accelerometer protocol *	Baseline 6 months	Only women	Middle age adults	Trained graduate students in psychology, public health or education
[52] Maki 2012	Older adults at risk of mental decline (n = 150) Mean age: 72	Community	RCT	Group meetings	Once a week for 90 minutes for 3 months.	Pedometer	Baseline 3 months	Both genders	Older adults	Registered physical trainers or health nurses
[53] Thomas 2012	Older adults (n = 399) Mean age: 72	Community centre for older people	RCT	Group-based, face-to-face counselling and advice Monthly telephone calls about 15 min	Once per month for 12 months with goal to reach 30 minutes three to five days per week	IPAQ * Pedometer	Baseline 6 months 12 months	Both genders	Older adults	Staff

List of abbreviation: CAPSPAQ: Cross Cultural Activity Participation Study Physical Activity Questionnaire; IPAQ: International Physical Activity Questionnaire; MLPAQ: Minnesota Leisure time Physical Activity Questionnaire; YPAS: Yale Physical Activity Survey; AIMS: Arthritis Impact Measurement Scale.

*Lay people* = people who received basic training to deliver the intervention, that is not part of their professional role.

*Professionals =* people who have previous education and work on behaviour change interventions as part of their professional.

* used as a basis for calculated effect sizes in meta-analysis.

### Meta-analysis

**Figure 2:**
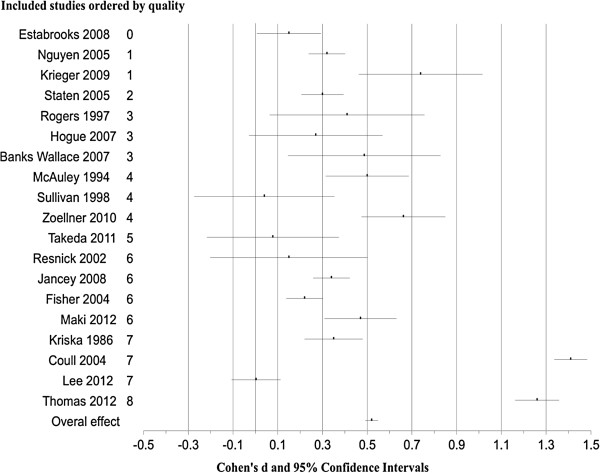
Forest plot showing changes in moderate physical activity for each study ordered by quality, as well as overall effect size (Cohen’s d) and 95% Confidence Intervals (CI).

A meta-analysis of the included studies indicated that interventions to promote walking in groups are efficacious at increasing physical activity (overall d = 0.52, 95%CI 0.32 to 0.71, N = 4752, k = 19, p < 0.0001). A forest plot showing physical activity effect sizes with 95% CI for each study ordered by quality assessment, is given in Figure 2. Fail-safe N was large: it would require that there would have to be an additional 753 studies showing a zero effect not included in the present study for the relationship between interventions and physical activity to become statistically non significant [34].

### Sensitivity analysis

Only one study [i.e. 53] of the 19 studies satisfied all of the quality criteria. There were twelve higher quality studies and seven lower quality studies (see Table 1). Overall, lower quality studies produced larger effect sizes (d = 0.59, 95%CI 0.28 to 0.91, n = 1610, k = 7, p < 0.0001) than higher quality studies (d = 0.49, 95%CI 0.24 to 0.75, n = 2962, k = 12, p < 0.0001) and that difference was statistical significant (z = 1.55, N = 4572, p = 0.05).

### Moderator analyses

The homogeneity of the studies was low (amount of variance explained by sampling error: 23.21%), indicating that other factors were causing substantial variation in effect sizes between studies. A summary of characteristics of included studies with theoretically relevant variables for conducting moderator analyses is provided in Table 2.

#### Outcome measures from baseline to six months vs over six months

Thirteen studies reported outcomes from baseline to 6 months and six studies reported outcomes over 6 months (see Table 2). Studies reporting short-term outcomes had overall smaller effect size (d = 0.45, 95%CI 0.25 to 0.65, n = 2992, k = 13, p < 0.0001) than studies reporting long-term outcomes (d = 0.66, 95%CI 0.22 to 1.10, n = 1580, k = 6, p < 0.001) and that difference was statistically significant (z = 3.32, N = 4572, p = 0.0004).

#### Only women vs both genders

They were six studies that targeted only women and the remaining thirteen targeted both genders (see Table 2). Interventions that targeted only women were found to have smaller effect size (d = 0.18, 95%CI from 0.03 to 0.33, n = 702, k = 6, p < 0.01) than interventions that targeted both genders (d = 0.61, 95%CI from 0.35 to 0.88, n = 3870, k = 13, p < 0.0001) and that difference was statistically significant (z = 5.12, N = 4572, p < 0.0001).

#### Younger adults vs older adults

They were 12 studies targeted adults up to 60 years and seven studies that targeted older adults (see Table 2). Interventions which target adults from 18 to 59 years old had smaller effect sizes (d = 0.48, 95%CI from 0.27 to 0.69, n = 2548, k = 12, p < 0.0001) than interventions that target older adults (d = 0.57 95%CI from 0.17 to 0.98, n = 2024, k = 7, p < 0.005), and that difference was statistically significant (z = 1.55, N = 4752, p = 0.05).

#### Delivered by lay people vs by professionals

There were eight studies where groups were led by lay people, who had received at least a basic training to become walk leaders. In three studies the researcher delivered an intervention, but did not accompany walkers while group walking. In seven studies groups were led by professionals (e.g. trained staff), and in one study was not clear who supervised the walking group (see Table 2). Interventions delivered by a lay person had a similar effect size (d = 0.52, 95%CI from 0.25 to 0.79, n = 2843, k = 8, p < 0.0001) to interventions delivered by professionals (d = 0.51, 95%CI from 0.23 to 0.79, n = 1729, k = 11, p < 0.0001). The difference in effect size estimates was not statistically significant (z = 0.158, N = 4572, p = 0.43).

## Discussion

### Summary of evidence

The meta-analysis of 19 studies with 4 572 participants showed that interventions to promote walking in groups are efficacious at increasing physical activity. The overall effect was of medium size (d = 0.52, p < 0.001) when all eligible studies were examined and slightly lower when only higher quality studies were included (d = 0.49, p < 0.001). Thus, the overall effect size found in this analysis might be an overestimate due to the inclusion of lower quality studies. Despite this, the overall effect size of the twelve higher quality studies was still highly statistically significant, which gives us more confidence that this finding is not a spurious finding due to poor quality studies.

### Strengths and limitations

A limitation of this systematic review was that several potentially eligible studies were identified, but no effect sizes could be calculated due to missing information. Even though authors of these studies were contacted, the necessary information was not provided by several authors. Thus, the true overall effect of the interventions to promote walking in groups might be slightly different from that estimated by the present study. However, there is every reason to believe that the overall effects would still be positive even if the results of these studies could have been included, as all studies from which we could not yield effect size estimates for this review reported positive changes in physical activity. Further, the large fail-safe N of 753 studies indicates that the finding of this meta-analysis is robust.

A related limitation might be that the searching of databases and selection of the studies was performed only by the first author due to resource limitations. This could have introduced bias into the selection of studies. However the large fail-safe N indicates that there would need to have been a substantial systematic bias in the selection of studies for the finding of positive effects of walking groups to be inaccurate.

Only studies reported in English language are reported, which limits the generalizability of the results of this review. Another issue was the limited generalizability of the study population. This was because the majority of the participants were white middle age women. No comparisons based on participants’ ethnicity or other possible confounders were performed, due to limited information provided in the primary studies. This limits our ability to draw conclusions about the effects of other characteristics of the study population on the outcome. We should therefore treat this evidence cautiously when translated in practice to different demographic groups.

This review is also limited by the heterogeneity of the studies included. In this review we conducted only a small number of moderator analyses, based on questions derived from the relevant literature. It seems likely that other factors which have not been explored in this review may account for the variance unexplained by these moderators, which future research should aim to identify (e.g. characteristics of participants at baseline, treatment of the control groups, quality of training of walk leaders). It is also worth noting that the moderator analyses reported were based on the entire set of studies, not just those of higher quality, due to the limited number of studies identified. Thus, it was possible that the results of some moderator analyses may have been affected by the moderating variables being associated with other variables responsible for heterogeneity of these studies (e.g. design, measures, etc.). However, the sensitivity analyses indicated that although the association between study quality and study physical activity effect size was significant (p = 0.05), it was not of sufficient size to be responsible for the results of the moderator analyses reported.

The outcome of this review might be limited by only five of the 19 studies having an objective measure of physical activity change (i.e. pedometer). However, two of them they were RCTs and all of them reported significant changes in physical activity, suggesting that the quality of outcome measure was not having a large effect on study outcome.

To our knowledge, this is the first systematic review and meta-analysis which has attempted to investigate whether interventions to promote walking in groups are efficacious and what are the characteristics of studies that are associated with being efficacious at promoting physical activity. The present review not only provides good evidence of behaviour change outcomes but also of the characteristics of these interventions that are associated larger changes in behaviour. Based on this, further recommendations on how walking in groups interventions could be more efficacious can be formed.

### Implications for policy and practice

Taking into consideration the health benefits of physical activity [8] and the benefits of group interventions to target more people than individually based interventions, it seems worth considering devoting resources to designing and implementing interventions to promote walking in groups.

Interventions that had measures from baseline up to six months had significantly lower effect sizes than interventions that had measures over six months. This finding is not consistent with previous research, which suggests that maintenance of behavioral changes in long-term is challenging [12,72]. The findings of the present review are therefore promising and support the idea that walking interventions which provide social support relationships for behaviour change may lead to greater maintenance of behaviour change [73]. Recent research using an objective measure of long-term maintenance at walking groups, suggested that participants maintain attendance at walking in groups for a long period of time when they have high self-efficacy and their outcomes expectations have been satisfied by the walking group intervention [74]. Thus future interventions might consider addressing participants’ self-efficacy and satisfying their outcome expectations in order to achieve long-term attendance at the behaviour change interventions.

Interventions that targeted both genders produced significantly higher effects on physical activity, compared with interventions that targeted only women. Unfortunately, we have no separate outcomes for men only to allow us comparison between women only and men only groups. Despite this, given the evidence about men’s low participation in walking groups [9], it is encouraging that interventions to promote walking in groups for both genders are more efficacious than interventions that target only women. Future interventions could assess men’s preferences for walking groups and tailor the interventions to their needs. This might attract more men at walking groups and promote the health benefits derived from walking groups to both genders.

Interventions that targeted older adults were found to be more efficacious than those that targeted younger adults. Although only seven of the 19 studies of this review targeted older adults they seem to be more efficacious than interventions that targeted younger adults. It has been estimated that more than half of the participants on led walks around UK are 65 and over [9], thus the results of this review indicates that this population benefits more from walking group interventions in terms of physical activity effects. This is a promising finding taking into account the growing proportion of people aged over 60 years and the challenge of public health to maximize the health and functional capacity of this population.

Interventions delivered by professionals were found not to have significant different effects than interventions delivered by lay people. This finding is in agreement with previous review on self-management interventions [75], suggesting that lay people when sufficiently trained can produce positive changes on walkers’ physical activity. Thus it might be worth training lay people on how to effectively deliver interventions to promote walking in groups.

### Unanswered questions

Taking into consideration the deficiencies of primary studies, there is a need for studies of best quality, namely RCTs with objective and long-term measures of behavioral outcomes.

It is important to highlight that the results of this review with meta-analysis present evidence about the *efficacy* of interventions to promote walking in groups but no clear evidence is being supported about the *effectiveness* of these interventions outside research studies. More pragmatic research, with more reliable ways of measuring outcome effects in the long-term and more rigorous designs is needed to give an answer to whether and how long-term behaviour change can be achieved in real world. Identifying those intervention components important for short-term and long-term behaviour change would be worth considering. Moreover, future studies should test whether these interventions are cost-effective.

## Endnote

^a^All data from original search was extracted by two independent researchers, but only first author extracted data from five additional studies added following an update and extended search after revision.

## Abbreviations

RCT: Randomized control trial; CAPSPAQ: Cross cultural activity participation study physical activity questionnaire; IPAQ: International physical activity questionnaire; MLPAQ: Minnesota leisure time physical activity questionnaire; YPAS: Yale physical activity survey; AIMS: Arthritis impact measurement scale; WfH: Walking for health; CI: Confidence intervals.

## Authors’ contributions

AK and DF designed the study. AK identified the eligibility criteria, conducted the searches, also data extraction and analyses, and drafted the manuscript. DF participated in decisions regarding eligibility criteria and supervised data analyses and drafting the manuscript. AT participated in its coordination and helped to draft the manuscript. All authors read and approved the final manuscript.

## Competing interest

The authors declare that they have no competing interest.

## Supplementary Material

Additional file 1 Search terms. Click here for file

Additional file 2 Coding frame used to code interventions to promote walking in group. Click here for file

Additional file 3 Sensitivity analysis, criteria for assessing quality. Click here for file
