# Supplementary material for: Do interventions to promote walking in groups increase physical activity? A meta-analysis
Source: Int J Behav Nutr Phys Act. 2013 Feb 6;10:18. doi: 10.1186/1479-5868-10-18 (PMC3585890; doi:10.1186/1479-5868-10-18)
Supplement: Additional file 1 — Search terms. [file 1479-5868-10-18-S1.doc]

**Appendix 1.** Search terms

Academic Search Complete, PsycINFO, Medline, CINAHL with full text, AMED, SprotDiscus search strategy

1. walking in groups/
2. group walking/
3. club walking/
4. led walk/
5. walking club/
6. group physical activity/
7. group exercise/
8. intervention/
9. pedometer intervention/
10. program/
11. or/1-7
12. or/1-7 and/ 8-10
13. walk* or physical activity and group* or club * or team* and intervention* or program*

Scopus search strategy

1. walking in groups/
2. group walking/
3. club walking/
4. walking club/
5. group physical activity/
6. group exercise/
7. intervention /
8. pedometer intervention/
9. interventions/
10. program/
11. or/ 1-6
12. or/ 1-6 and/ 7-10
