# Supplementary material for: Do interventions to promote walking in groups increase physical activity? A meta-analysis
Source: Int J Behav Nutr Phys Act. 2013 Feb 6;10:18. doi: 10.1186/1479-5868-10-18 (PMC3585890; doi:10.1186/1479-5868-10-18)
Supplement: Additional file 2 — Coding frame used to code interventions to promote walking in group. [file 1479-5868-10-18-S2.doc]

**Appendix 2.** Coding frame used to code interventions to promote walking in group

A draft coding frame was piloted on a few studies before the final coding frame (provided below) was produced.

| **Date of data extraction**: | | | | | | | | | | | | | | | | | | | | | | | | | | | | | | | | | | | |
| --- | --- | --- | --- | --- | --- | --- | --- | --- | --- | --- | --- | --- | --- | --- | --- | --- | --- | --- | --- | --- | --- | --- | --- | --- | --- | --- | --- | --- | --- | --- | --- | --- | --- | --- | --- |
| **Name of reviewer**: | | | | | | | | | | | | | | | | | | | | | | | | | | | | | | | | | | | |
| **Study details**: *(please state all documents found to describe same study)* | | | | | | | | | | | | | | | | | | | | | | | | | | | | | | | | | | | |
| **Authors:** | | | | | | | | | | | | | | | | | | | | | | | | | | | | | | | | | | | |
|  | | | | | | | | | | | | | | | | | | | | | | | | | | | | | | | | | | | |
| **Objectives of the intervention** *(please describe as in articles)* | | | | | | | | | | | | | | | | | | | | | | | | | | | | | | | | | | | |
|  | | | | | | | | | | | | | | | | | | | | | | | | | | | | | | | | | | | |
| **General Comments/ Notes** | | | | | | | | | | | | | | | | | | | | | | | | | | | | | | | | | | | |
|  | | | | | | | | | | | | | | | | | | | | | | | | | | | | | | | | | | | |
| **Control group** | | | | **Group 1** | | | | | **Group 2** | | | | | | | | | | | | **Overall** | | | | | | | | | | | | | | |
| **Description** | | | | **Description** | | | | | **Description** | | | | | | | | | | | | **Description** | | | | | | | | | | | | | | |
|  | | | |  | | | | |  | | | | | | | | | | | |  | | | | | | | | | | | | | | |
|  | | | | | | | | | | | | | | | | | | | | | | | | | | | | | | | | | | | |
| **Participant characteristics** | | | | | | | | | | | | | | | | | | | | | | | | | | | | | | | | | | | |
|  | | | | | **Control group** | | | | **Group 1** | | | | | | | | **Group 2** | | | | | | | | **Overall** | | | | | | | | | | |
| No. of included participants (number receiving intervention) | | | | |  | | | |  | | | | | | | |  | | | | | | | |  | | | | | | | | | | |
| Mean age (M)  St. Deviation (SD) | | | | |  | | | |  | | | | | | | |  | | | | | | | |  | | | | | | | | | | |
| No. of males (No.) | | | | |  | | | |  | | | | | | | |  | | | | | | | |  | | | | | | | | | | |
| No. of females (No.) | | | | |  | | | |  | | | | | | | |  | | | | | | | |  | | | | | | | | | | |
| Ethnicity (No.) | | | | |  | | | |  | | | | | | | |  | | | | | | | |  | | | | | | | | | | |
| Education | | | | |  | | | |  | | | | | | | |  | | | | | | | |  | | | | | | | | | | |
|  | | | | | | | | | | | | | | | | | | | | | | | | | | | | | | | | | | | |
| **Group characteristics** *(please tick or write as appropriate)* | | | | | | | | | | | | | | | | | | | | | | | | | | | | | | | | | | | |
| Chronic diseases (e.g. cardiovascular diseases, diabetes, cancer) | | | | | | | | | | | | | | | | | | | | | | | | | | | | | | |  | | | | |
| Healthy adults | | | | | | | | | | | | | | | | | | | | | | | | | | | | | | |  | | | | |
| Ethnic minority groups | | | | | | | | | | | | | | | | | | | | | | | | | | | | | | |  | | | | |
| Only women | | | | | | | | | | | | | | | | | | | | | | | | | | | | | | |  | | | | |
| Older adults | | | | | | | | | | | | | | | | | | | | | | | | | | | | | | |  | | | | |
| Other (specify) | | | | | | | | | | | | | | | | | | | | | | | | | | | | | | |  | | | | |
|  | | | | | | | | | | | | | | | | | | | | | | | | | | | | | | |  | | | | |
|  | | | | | | | | | | | | | | | | | | | | | | | | | | | | | | | | | | | |
| **Inclusion criteria** (*please describe as in articles*) | | | | | | | | | | | | | | | | | | | | | | | | | | | | | | | | | | | |
|  | | | | | | | | | | | | | | | | | | | | | | | | | | | | | | | | | | | |
|  | | | | | | | | | | | | | | | | | | | | | | | | | | | | | | | | | | | |
| **Intervention characteristics** (*please highlight or write as appropriate*) | | | | | | | | | | | | | | | | | | | | | | | | | | | | | | | | | | | |
| **Focus of intervention:**  Physical activity (specify type of PA)  Multi component   - Diet - Smoking - Other (specify) | | | | | | | | | | | | | | | | | | | | | | | | | | | | | | | | | | | |
| **Main outcome measure:**  Please specify:   - frequency - distance - duration - other (specify) | | | | | | | | | | | | | | | | | | | | | | | | | | | | | | | | | | | |
| **Setting:**   - Community - Workplace - College/University - GP Surgery - Community Centre - By post - Other (please specify) | | | | | | | | | | | | | | | | | | | | | | | | | | | | | | | | | | | |
| **Delivered by**:   - Lay expert - Walk-leader - Researcher - Nurse - Physiotherapists - GP - Peers - Not stated - Health and Fitness Professional - Other (Please specify) | | | | | | | | | | | | | | | | | | | | | | | | | | | | | | | | | | | |
| **Delivery mode:**   - Training session - Discussion Group - Web-based - Telephone - Self-help manuals - Mass media - Postal - Not stated - Other (Please specify) | | | | | | | | | | | | | | | | | | | | | | | | | | | | | | | | | | | |
| **Duration of intervention:** | | | | | | | | | | | | | | | | | | | | | | | | | | | | | | | | | | | |
| **Number of intervention contact sessions:** | | | | | | | | | | | | | | | | | | | | | | | | | | | | | | | | | | | |
| **Timing of interventions sessions:** | | | | | | | | | | | | | | | | | | | | | | | | | | | | | | | | | | | |
| **Design of study:**   - Experiment (Randomised) - Experiment (Non-randomised) - Quasi-experimental (Already have naturally distinct groups, e.g. two workplaces) - Pre - post study - Unsure/Not stated - Other (*please state*) | | | | | | | | | | | | | | | | | | | | | | | | | | | | | | | | | | | |
| **Unit of allocation:**   - Individual - Group | | | | | | | **Allocation by:**   - Experimenter - Self-selected | | | | | | | | | | | **Type of allocation:**   - Simple - Group - Stratified - Other (specify) | | | | | | | | | | | | | | | | | |
| **Randomization:** | | | | | | | | | | | | | | | | | | | | | | | | | | | | | | | | | | | |
| **Where is the intervention described?**   - All in this paper - All described elsewhere - Additional information described elsewhere (please note) - Based on another study, described elsewhere | | | | | | | | | | | | | | | | | | | | | | | | | | | | | | | | | | | |
|  | | | | | | | | | | | | | | | | | | | | | | | | | | | | | | | | | | | |
| **Techniques***(please describe as in article- add more columns if appropriate)* | | | | | | | | | | | | | | | | | | | | | | | | | | | | | | | | | | | |
| **Implemented to walkers** | | | | | | | | | | | | | | | | | | | | | | | | | | | | | | | | | | | |
| **N** | **Technique as described in the article** | | | | | | | | | **Control** | | | | | | **Group 1** | | | **Group 2** | | | | | **Taxonomy** | | | | | | | | **Details** | | | |
|  |  | | | | | | | | |  | | | | | |  | | |  | | | | |  | | | | | | | |  | | | |
|  |  | | | | | | | | |  | | | | | |  | | |  | | | | |  | | | | | | | |  | | | |
|  |  | | | | | | | | |  | | | | | |  | | |  | | | | |  | | | | | | | |  | | | |
|  |  | | | | | | | | |  | | | | | |  | | |  | | | | |  | | | | | | | |  | | | |
|  |  | | | | | | | | |  | | | | | |  | | |  | | | | |  | | | | | | | |  | | | |
|  |  | | | | | | | | |  | | | | | |  | | |  | | | | |  | | | | | | | |  | | | |
|  |  | | | | | | | | |  | | | | | |  | | |  | | | | |  | | | | | | | |  | | | |
|  |  | | | | | | | | |  | | | | | |  | | |  | | | | |  | | | | | | | |  | | | |
| **Implemented to leaders** | | | | | | | | | | | | | | | | | | | | | | | | | | | | | | | | | | | |
| **N** | **Technique as described in the article** | | | | | | | | | **Control** | | | | | | **Group 1** | | | **Group 2** | | | | | **Taxonomy** | | | | | | | | **Details** | | | |
|  |  | | | | | | | | |  | | | | | |  | | |  | | | | |  | | | | | | | |  | | | |
|  |  | | | | | | | | |  | | | | | |  | | |  | | | | |  | | | | | | | |  | | | |
|  |  | | | | | | | | |  | | | | | |  | | |  | | | | |  | | | | | | | |  | | | |
|  |  | | | | | | | | |  | | | | | |  | | |  | | | | |  | | | | | | | |  | | | |
|  |  | | | | | | | | |  | | | | | |  | | |  | | | | |  | | | | | | | |  | | | |
|  |  | | | | | | | | |  | | | | | |  | | |  | | | | |  | | | | | | | |  | | | |
|  | | | | | | | | | | | | | | | | | | | | | | | | | | | | | | | | | | | |
| **Theoretical background** *(please tick, highlight or write as appropriate)* | | | | | | | | | | | | | | | | | | | | | | | | | | | | | | | | | | | |
| The theoretical constructs were targeted by intervention techniques | | theory specified in introduction to journal article | | | | | | | | | | | | | | | | | | | | | | | | | | | | | | |  | | |
|  |  | explicit description of how theory-based interventions targeted psychological constructs | | | | | | | | | | | | | | | | | | | | | | | | | | | | | | |  | | |
| Theoretical constructs were measured | | all theoretical constructs were measured | | | | | | | | | | | | | | | | | | | | | | | | | | | | | | |  | | |
|  |  | at least one theoretical construct was measured | | | | | | | | | | | | | | | | | | | | | | | | | | | | | | |  | | |
| Mediation effects were tested and discussed on how the intervention brought about behaviour change | | mediation of any/all theoretical constructs was measured | | | | | | | | | | | | | | | | | | | | | | | | | | | | | | |  | | |
|  |  | findings were explained in relation to theory, or theories | | | | | | | | | | | | | | | | | | | | | | | | | | | | | | |  | | |
| **Specify (which theory has been used)**   - TTM - TPB - SCT - Self-efficacy - other (please specify) | | | | | | | | | | | | | | | | | | | | | | | | | | | | | | | | | | | |
|  | | | | | | | | | | | | | | | | | | | | | | | | | | | | | | | | | | | |
| **Recruitment** *(please describe as in article)* | | | | | | | | | | | | | | | | | | | | | | | | | | | | | | | | | | | |
|  | | | | | | | | | | | | | | | | | | | | | | | | | | | | | | | | | | | |
|  | | | | | | | | | | | | | | | | | | | | | | | | | | | | | | | | | | | |
| **Needs assessment** *(please tick, highlight or write as appropriate)* | | | | | | | | | | | | | | | | | | | | | | | | | | | | | | | | | | | |
| **Needs assessment pre intervention**  *Details:* | | | | | | | | | | | | | | | | | | | | | | | | | | | **Yes** | | | | | | |  | |
|  |  |  |  |  |  |  |  |  |  |  |  |  |  |  |  |  |  |  |  |  |  |  |  |  |  |  | **No** | | | | | | |  | |
|  |  |  |  |  |  |  |  |  |  |  |  |  |  |  |  |  |  |  |  |  |  |  |  |  |  |  | **Unclear** | | | | | | |  | |
| If yes, please specify:   - Focus groups - Face to face interview - Quantitative measurements (please specify) - Other | | | | | | | | | | | | | | | | | | | | | | | | | | | | | | | | | | | |
| **Needs assessment post intervention/ whether the intervention met participants needs** | | | | | | | | | | | | | | | | | | | | | | | | | | | | | **Yes** | | | | | |  |
|  |  |  |  |  |  |  |  |  |  |  |  |  |  |  |  |  |  |  |  |  |  |  |  |  |  |  |  |  | **No** | | | | | |  |
|  |  |  |  |  |  |  |  |  |  |  |  |  |  |  |  |  |  |  |  |  |  |  |  |  |  |  |  |  | **Unclear** | | | | | |  |
| If yes, please specify:   - Focus groups - Face to face interview - Quantitative measurements (please specify) - Other | | | | | | | | | | | | | | | | | | | | | | | | | | | | | | | | | | | |
|  | | | | | | | | | | | | | | | | | | | | | | | | | | | | | | | | | | | |
| **Measurement tool for physical activity** *(please highlight or write as appropriate)* | | | | | | | | | | | | | | | | | | | | | | | | | | | | | | | | | | | |
| - Pedometers - Accelerometer - Questionnaires (specify) - Diaries/ calendars - Other (please note) | | | | | | | | | | | | | | | | | | | | | | | | | | | | | | | | | | | |
| Validated measurement tool | | | | | | | | | | | | Reliability α’s reported | | | | | | | | | | | | | | | | | | | | | | | |
| Single item measure | | | | | | | | | | | | | | | | | | | | | | | | | | | | | | | | | | | |
| Multiple item measure:  Number of items | | | | | | | | | | | | | Reliability α’s reported | | | | | | | | | | | | | | | | | | | | | | |
| Not sure | | | | | | | | | | | | | | | | | | | | | | | | | | | | | | | | | | | |
| Details not given | | | | | | | | | | | | | | | | | | | | | | | | | | | | | | | | | | | |
|  | | | | | | | | | | | | | | | | | | | | | | | | | | | | | | | | | | | |
| **Analysis** | | | | | | | | | | | | | | | | | | | | | | | | | | | | | | | | | | | |
| **At baseline** | | | | | | | | | | | | | | | | | | | | | | | | | | | | | | | | | | | |
| **At follow up** | | | | | | | | | | | | | | | | | | | | | | | | | | | | | | | | | | | |
| **Time point of follow up measures**  Time 1=  Time 2=  Time 3= | | | | | | | | | | | | | | | | | | | | | | | | | | | | | | | | | | | |
| **Time point of main analysis** | | | | | | | | | | | | | | | | | | | | | | | | | | | | | | | | | | | |
| **Participants included in the analysis** | | | | | | | | | | | | | | | | | | | | | | | | | | | | | | | | | | | |
| **Uptake** | | | | | | | | | | | | | | | | | | | | | | | | | | | | | | | | | | | |
|  | | | | | | | | **Group 1** | | | | | | **Group 2** | | | | | | | | **Group 3** | | | | **Overall** | | | | | | | | | |
| Number eligible for the intervention  (e.g. number of residents in community based interventions) | | | | | | | |  | | | | | |  | | | | | | | |  | | | |  | | | | | | | | | |
| Number approached | | | | | | | |  | | | | | |  | | | | | | | |  | | | |  | | | | | | | | | |
| Responded to advertisement  /Expressed interest in taking part | | | | | | | |  | | | | | |  | | | | | | | |  | | | |  | | | | | | | | | |
| Eligible to take part | | | | | | | |  | | | | | |  | | | | | | | |  | | | |  | | | | | | | | | |
| Informed consent given | | | | | | | |  | | | | | |  | | | | | | | |  | | | |  | | | | | | | | | |
| Baseline | | | | | | | |  | | | | | |  | | | | | | | |  | | | |  | | | | | | | | | |
| Retention | | | | | | | | | | | | | | | | | | | | | | | | | | | | | | | | | | | |
| Retention rate/number lost to follow up (number at each stage) | | | | | | | |  | | | | | |  | | | | | | | |  | | | |  | | | | | | | | | |
| Time 1 | | | | | | | |  | | | | | |  | | | | | | | |  | | | |  | | | | | | | | | |
| Time 2 | | | | | | | |  | | | | | |  | | | | | | | |  | | | |  | | | | | | | | | |
| Time 3 | | | | | | | |  | | | | | |  | | | | | | | |  | | | |  | | | | | | | | | |
| Drop out | | | | | | | | | | | | | | | | | | | | | | | | | | | | | | | | | | | |
| **Reasons for dropping out**:   - Excluded by researcher - Non-completion of measures - medical conditions - other commitments (work, family etc) - intervention design wasn’t convenient (eg. time, location etc) - not motivated - Outliers - Other - not mentioned | | | | | | | | | | | | | | | | | | | | | | | | | | | | | | | | | | | |
| **How was attrition dealt with**? (*please describe as in article*) | | | | | | | | | | | | | | | | | | | | | | | | | | | | | | | | | | | |
| **A priori power calculation**  *Details:* | | | | | | | | | | | | | | | | | | | | | | | | | **Yes** | | | | | | | |  | | |
|  |  |  |  |  |  |  |  |  |  |  |  |  |  |  |  |  |  |  |  |  |  |  |  |  | **No** | | | | | | | |  | | |
|  |  |  |  |  |  |  |  |  |  |  |  |  |  |  |  |  |  |  |  |  |  |  |  |  | **Unclear** | | | | | | | |  | | |
|  | | | | | | | | | | | | | | | | | | | | | | | | | | | | | | | | | | | |
| **Results** | | | | | | | | | | | | | | | | | | | | | | | | | | | | | | | | | | | |
|  | | | | | | | | | | | | | | | **Between group differences** | | | | | | | | | | | | | | | | | | | | |
|  | | | **Control**  **M(SD)** | | | **Group 1**  **M(SD)** | | | **Group 2**  **M(SD)** | | | | | | **Effect size** | | | | | | | | **P-value** | | | | | **Test statistics** | | | | | | | |
| **Baseline**  Physical activity | | |  | | |  | | |  | | | | | |  | | | | | | | |  | | | | |  | | | | | | | |
| **Time point 1**  Physical activity | | |  | | |  | | |  | | | | | |  | | | | | | | |  | | | | |  | | | | | | | |
| **Time point 2**  Physical activity | | |  | | |  | | |  | | | | | |  | | | | | | | |  | | | | |  | | | | | | | |
| **Time point 3**  Physical activity | | |  | | |  | | |  | | | | | |  | | | | | | | |  | | | | |  | | | | | | | |
|  | | | | | | | | | | | | | | | | | | | | | | | | | | | | | | | | | | | |
| **Other results (please state)** | | | | | | | | | | | | | | | | | | | | | | | | | | | | | | | | | | | |
|  | | | | | | | | | | | | | | | | | | | | | | | | | | | | | | | | | | | |
|  | | | | | | | | | | | | | | | | | | | | | | | | | | | | | | | | | | | |
| **Assessment of quality / 2 criteria** *(please highlight)* | | | | | | | | | | | | | | | | | | | | | | | | | | | | | | | | | | | |
| Informed consent | | | | | | | | | | | **Yes** | | | | | | | | | **No** | | | | | | | | | | **Unclear** | | | | | |
| Ethical approval | | | | | | | | | | | **yes** | | | | | | | | | **No** | | | | | | | | | | **Unclear** | | | | | |
