# Supplementary material for: Do interventions to promote walking in groups increase physical activity? A meta-analysis
Source: Int J Behav Nutr Phys Act. 2013 Feb 6;10:18. doi: 10.1186/1479-5868-10-18 (PMC3585890; doi:10.1186/1479-5868-10-18)
Supplement: Additional file 3 — Sensitivity analysis, criteria for assessing quality. [file 1479-5868-10-18-S3.doc]

**Appendix 3.** Sensitivity analysis, criteria for assessing quality

Assessment of the quality of the included studies was made using the guide for review authors on assessing study quality by Cochrane Consumers and Communication Review Group (Ryan et al. 2011). If studies satisfied each of the following criteria, were awarded a point “*yes*”, if not they were not given any point “*no*”. In case where studies report the name of any of the following criteria but did not provide any further details on the process followed to satisfy the criteria, then it was assumed that the adequacy of the description was “*unclear”* and no points were given.

- Randomization: whether there was sufficient description of a randomization process. Studies that were awarded a point only if there was a description that produced a non-predictable assignment pattern. For example computer generated random numbers, random number tables, coin toss (for a trial with two groups) or die toss (when trial had more than two groups). Studies were not awarded a point when used inadequate approaches, like birth dates, week days, case record numbers, and alternation.
- Allocation concealment: when the researcher is not aware of the characteristics of participants recruited into trial before the allocation of participants to experimental groups. For example the allocation of the participants in the trial has been done from a central computer away from researchers, from on-site computer from which assignment can only be determined after entering the participants’ data, from re-numbered or coded identical containers administered serially to participants, using serially (sequentially) numbered, using opaque sealed envelopes or if administered by a different person to the one who generated the allocation scheme. Inadequate allocation was selected if the following were used: any ‘open methods’ (ie. transparent before allocation) e.g. lists on notice boards, open lists, open envelopes, non-opaque envelopes, odd or even date or medical record number, dates of birth or days of week, alternation.
- Blinding: researchers allocating participants did not know which participant belongs to which experimental group, from participants’ allocation until the analysis and interpretation of the research evidence. Whereas allocation concealment is achieved when the randomisation sequence is concealed *before* and up until the point at which people are allocated to groups, blinding refers to measures that are taken *after* people have been assigned to groups. This means that no-one knows which participant belongs to the different groups throughout the course of the study.
- Baseline comparability: whether researchers did statistical tests to examine how demographic and psychological variables differed before randomization and include these as a covariance in analysis of main outcome.
- Follow up: whether the number of the follow up participants was at least 80% of the baseline.
- Validation of measures: whether the measures used were validated or not. A credit was given to studies when they mentioned the validity of the measures used, had references with the validation process or when validation process could be obtained via search in the databases.
- Ethics approval: whether the studies was approved by an appropriately constituted Human Ethics Review Committee.
- Informed consent: whether participants provided informed consent to their participation in the study

**References**

Ryan, R., Hill, S., Prictor, M. & McKenzie, J. 2011*, Cochrane Consumers and Communication Review Group. Study Quality Guide.* <http://www.latrobe.edu.au/chcp/assets/downloads/StudyQualityGuide_May2011.pdf> [accessed May 2011]
